# Eating Behaviour among Multi-Ethnic Adolescents in a Middle-Income Country as Measured by the Self-Reported Children’s Eating Behaviour Questionnaire

**DOI:** 10.1371/journal.pone.0082885

**Published:** 2013-12-05

**Authors:** Debbie Ann Loh, Foong Ming Moy, Nur Lisa Zaharan, Zahurin Mohamed

**Affiliations:** 1 Julius Centre University of Malaya, Department of Social and Preventive Medicine, Faculty of Medicine, University of Malaya, Kuala Lumpur, Malaysia; 2 Department of Pharmacology, Faculty of Medicine, University of Malaya, Kuala Lumpur, Malaysia; University of Barcelona, Faculty of Biology, Spain

## Abstract

**Background:**

Escalating weight gain among the Malaysian paediatric population necessitates identifying modifiable behaviours in the obesity pathway.

**Objectives:**

This study describes the adaptation and validation of the Children’s Eating Behaviour Questionnaire (CEBQ) as a self-report for adolescents, investigates gender and ethnic differences in eating behaviour and examines associations between eating behaviour and body mass index (BMI) z-scores among multi-ethnic Malaysian adolescents.

**Methodology:**

This two-phase study involved validation of the Malay self-reported CEBQ in Phase 1 (n = 362). Principal Axis Factoring with Promax rotation, confirmatory factor analysis and reliability tests were performed. In Phase 2, adolescents completed the questionnaire (n = 646). Weight and height were measured. Gender and ethnic differences in eating behaviour were investigated. Associations between eating behaviour and BMI z-scores were examined with complex samples general linear model (GLM) analyses, adjusted for gender, ethnicity and maternal educational level.

**Results:**

Exploratory factor analysis revealed a 35-item, 9-factor structure with ‘food fussiness’ scale split into two. In confirmatory factor analysis, a 30-item, 8-factor structure yielded an improved model fit. Reliability estimates of the eight factors were acceptable. Eating behaviours did not differ between genders. Malay adolescents reported higher Food Responsiveness, Enjoyment of Food, Emotional Overeating, Slowness in Eating, Emotional Undereating and Food Fussiness 1 scores (p<0.05) compared to Chinese and Indians. A significant negative association was observed between BMI z-scores and Food Fussiness 1 (‘dislike towards food’) when adjusted for confounders.

**Conclusion:**

Although CEBQ is a valuable psychometric instrument, adjustments were required due to age and cultural differences in our sample. With the self-report, our findings present that gender, ethnic and weight status influenced eating behaviours. Obese adolescents were found to display a lack of dislike towards food. Future longitudinal and qualitative studies are warranted to further understand behavioural phenotypes of obesity to guide prevention and intervention strategies.

## Introduction

Countries across the spectrum of development are grappling with burgeoning rates of childhood obesity [1,2] although recent evidence of plateauing prevalence in few countries has emerged [3]. Chronic obesity-related diseases previously diagnosed among adults have now emerged among paediatric populations [1,4,5]. Malaysia, a developing, middle-income and ethnically diverse country situated in South-East Asia, is likewise experiencing a concomitant rise in paediatric obesity [6,7]. The National Health and Morbidity Survey (NHMS IV) in 2011 reported that on a national level, 6.1% of Malaysian children and adolescents aged below 18 years were obese [8]. 

While eating is a necessity for survival, food intake beyond energy requirements in today’s ‘obesogenic’ environment is common, leading to consumption largely stimulated by pleasure (‘hedonic hunger’) rather than physiological hunger [9]. Interestingly, not all individuals become obese, some remain lean despite increased food intake. Differences in heritable neurobehavioural and psychological traits affecting eating behaviours may explain a greater predisposition or resistance to weight gain observed among individuals [10]. 

Eating behaviour is influenced by both internal and external determinants including food availability, knowledge, attitudes, emotional state and experiences of the individual [11]. Profiles of eating behaviours that predict weight status is a growing area of research. Although experimental laboratory studies are objective measures of eating behaviour, it is often impractical for large-scale research due to external factors present during the test, a limited number of participants allowed and the cost of setup [12]. The development of several psychometric tools has made epidemiological studies on eating behaviour possible. The most comprehensive tool currently identified is the Children’s Eating Behaviour Questionnaire (CEBQ) developed by Wardle and colleagues [13].

The CEBQ is a 35-item questionnaire, originally designed as a parent-report to measure eating behaviour traits related to obesity risk among children aged below 12 years old. All items were developed from a combination of literature review and interviews with parents [13]. The CEBQ contains eight dimensions of eating styles; four subscales that measure food-approach behaviours [Food Responsiveness (FR), Enjoyment of Food (EF), Emotional Overeating (EOE), Desire to Drink (DD)] and another four subscales that reflect food-avoidant behaviours [Satiety Responsiveness (SR), Slowness in Eating (SE), Emotional Undereating (EUE) and Food Fussiness (FF)]. 

FR reflects response to environmental food cues while EF refers to a general interest in food, traits strongly observed in overweight or obese children [12,13]. EOE and EUE represent fluctuating food intake during negative emotional states. BMI in children has been positively associated with EOE and negatively associated with EUE [14-17]. DD represents the desire to drink liquids, particularly sugar-sweetened beverages (SSBs) [13]. Frequent consumption of SSBs have shown strong positive associations with BMI [18]. SR reflects the ability to regulate food intake according to internal satiety cues. Overweight and obese individuals tend to allow external food cues to override internal satiety signals [12]. A reduced eating speed due to a lack of enjoyment and decreased interest in food characterises SE. Obese children tend to eat faster towards the end of a meal [19]. FF is described as being highly selective of foods resulting in a limited variety of foods consumed. To date, associations between fussy eaters and BMI among children have been inconclusive [14,15,17]. 

The CEBQ has demonstrated good internal consistency, test-retest reliability and has been validated in the United Kingdom, Portugal, Chile, the Netherlands, Sweden, China and Canada; investigating eating behaviours of toddlers and children [13-16,20-22]. This instrument has been successfully employed as a parent-report in various studies including assessing the continuity and stability in eating behaviour of children, appetite preference in children of lean and obese parents, investigating relationships between temperament and eating behaviours and associations between appetitive traits with weight and dietary intake [23-26]. As such, this study intended to extend the CEBQ to adolescents as a self-report.

Food habits, eating patterns, dietary intake and risk of eating disorders among children and adolescents of varying weight status across Malaysia have been extensively studied [27-29], mirroring the unhealthy habits of their Western counterparts. However, investigations focused on appetitive traits associated with weight gain is unexplored amongst Malaysian children or adolescents. Studies in different cultures are necessary to provide insights into the similarities and differences inherent across cultural borders with regards to eating behaviours related to obesity. This present study describes the adaptation of the CEBQ as a self-report among adolescents in the local setting. The factor structure and psychometric properties of the Malay version of the self-reported CEBQ were examined. Gender and ethnic differences in eating behaviour were explored and associations between eating behaviour and BMI z-scores among multi-ethnic adolescents were further investigated. 

## Methods

### Study design

This cross-sectional study was conducted in two phases: Phase 1 – validation study and Phase 2 – association study.

### Study participants

Thirteen year old, Malaysian adolescents in Form One who were literate in Bahasa Malaysia (Malay language) from government-funded national secondary schools in Kuala Lumpur were included in this study. Vernacular, vocational, boarding, religious, private schools and schools for the handicapped were excluded. Adolescents with chronic diseases, on long term medication, weight gain supplements or on restricted diets based on parent-report were excluded from this study.

Ethics clearance was obtained from the Medical Ethics Committee of University of Malaya Medical Centre (UMMC) (Reference Number: MEC 896.123). Written approval to conduct the study was obtained from the Ministry of Education, Malaysia, the Federal Territory of Kuala Lumpur Education Department and the respective school principals. Written informed consent was obtained from all parents of participating adolescents prior to the study.

### Sample size calculation

In Phase 1, construct validity using factor analysis was conducted following the 10 to 1 item-subject ratio as recommended by Hair et al. [30]. Mutually exclusive samples with a minimum of 300 subjects were required for exploratory factor analysis (EFA) and confirmatory factor analysis (CFA) [31,32]. 

In Phase 2, sample size was determined using Open Epi version 2.3.1 software [33]. Confidence level was set at 95%, power of 80% with a prevalence rate of overweight of 0.20 based on a study by Rampal et al. [7] among secondary school students in Klang district in the neighbouring state of Selangor. The minimum sample size required was 426. The final sample size determined to accommodate a non-response rate of 30% was 554 subjects.

### Sampling method

In Phase 1, a total of 386 participants were recruited from two conveniently selected secondary schools in Kuala Lumpur. A subsample of 151 participants was selected to assess the test-retest reliability (7 day interval) of the self-administered questionnaire.

In Phase 2, a total of 652 participants were selected via multi-stage sampling from 23 randomly selected government-funded national secondary schools from different geographical zones and school streams (co-educational, male and female schools) in Kuala Lumpur. The samples recruited in both phases were independent of each other, that is, schools involved in Phase 1 were mutually exclusive from participating schools in Phase 2. 

### Study instruments

Prior permission was obtained from the original author, Professor Jane Wardle to use the study instrument. The CEBQ is a 35-item questionnaire, originally designed as a multi-dimensional parent-report to measure eating behaviour traits related to risk of obesity in toddlers and children below age 12. An instrument to assess obesity-related appetitive traits among adolescents was lacking in the local setting, therefore, a self-report was required for this older age group.

The self-reported CEBQ was developed with the original author and her team of experts to extend its use among adolescents. Review and comments from Wardle and her colleagues constituted the content validity of the self-reported CEBQ. Age and cultural differences were considered when modifications were required. Firstly, the third person pronoun, ‘My child’ (parent-report) was changed to the first person pronoun, ‘I’ (self-report) for all items. Secondly, ‘drinks’ for items 6, 29 and 31 (DD) were specified as ‘soft drinks’ since consumption of SSBs has strong associations with weight gain in children and adolescents [18]. While alcohol abuse among adolescents may be an issue among Western adolescents, this was not included in our study as alcohol is religiously prohibited among Malays and banned in schools. The specific duration for finishing meals in item 18 (SE) was omitted. The original statement ‘My child takes more than 30 minutes to finish a meal’ was changed to ‘I take a long time to finish a meal’ since with adolescents, the duration of meal depends on the type of meal, whether a quick sandwich on-the-go or dinner party with friends. Item 4 (SR) which was originally ‘My child finishes his/her meal quickly’ was rephrased to ‘I finish my meals more quickly than others’. 

Several semantic changes were made for relevant items to reflect autonomous eating and drinking among adolescents. For example, Item 6 (DD) was changed from ‘My child is always asking for a drink’ to ‘I always want soft drinks’, item 14 (FR) was changed from ‘If allowed to, my child would eat too much’ to ‘I tend to eat more than I should’ and item 34 (FR) from ‘Given the chance, my child would always have food in his/her mouth’ to ‘Given the chance, I would always have something to munch on’.

Similar to the original structure, the self-reported CEBQ had 35 items with response options using a five-point Likert scale (1 = never, 2 = rarely, 3 = sometimes, 4 = often and 5 = always). Each of the eight subscales contained 3 to 6 items. Items 3 (SR), 4 (SE), 10 (FF), 16 (FF) and 32 (FF) were reverse-scored items [13].

#### Translations

Forward and backward translation of the questionnaire was conducted. Two bilingual, professional translators performed translations of the English self-reported CEBQ into the target language (hereafter referred to as Malay). Both the drafts were reviewed and reconciled to a single Malay version. The backward translation from Malay to English was undertaken by another two bilingual translators, fluent in both languages. These individuals were blinded to the original English version. Discrepancies that arose were discussed to ensure that the Malay version reflected the meaning of the original self-reported CEBQ. A pre-test was conducted among a mixed-gender group of 13 year old adolescents (n = 8) from the three major ethnic groups in Malaysia (Malays, Chinese and Indians) with different socioeconomic backgrounds. Face-to-face interviews facilitated discussion to assess the clarity, wording and refine the questionnaire to maximise response and minimise respondent burden. 

### Study procedures

**Figure 1:**
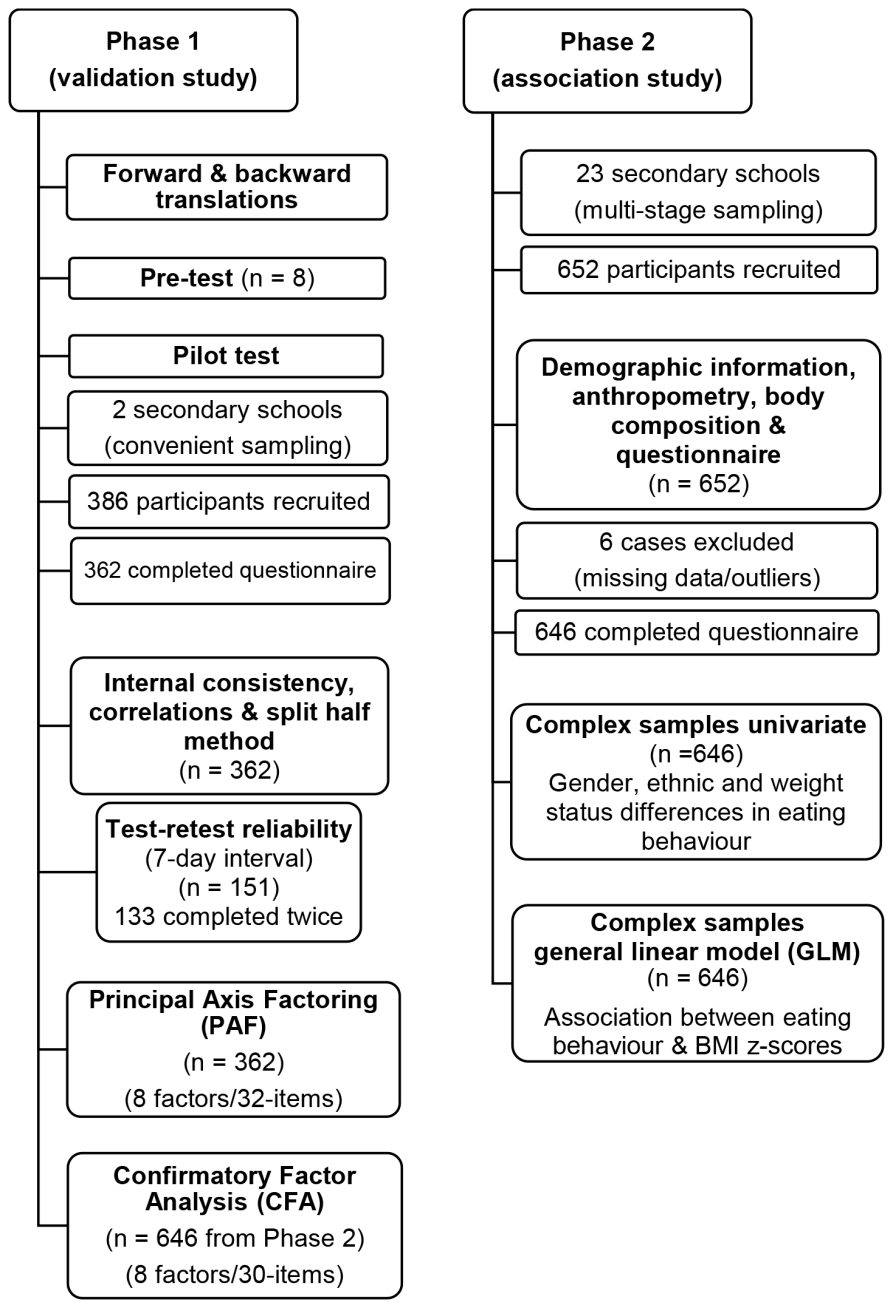
Flowchart of study procedures.

An overview of the study procedures is illustrated in Figure 1. In Phase 1, the final version of the Malay self-reported CEBQ was distributed to 386 Form One students in two conveniently selected government-funded secondary schools in Kuala Lumpur. A total of 362 participants completed the self-administered questionnaire in school without the guidance of the researcher or teachers, yielding a response rate of 93.8%. 

A test-retest study was conducted among a subsample of 151 students from one school that consented. The questionnaire was self-administered twice within a 7 day interval. A total of 133 adolescents completed the questionnaire on both occasions. 

In Phase 2, a total of 652 adolescents aged 13 years were recruited via multi-stage sampling from 23 government-funded national secondary schools in Kuala Lumpur. The Malay self-reported CEBQ was self-administered by the adolescents with 646 completed questionnaires returned (99.08%). Parents were requested to provide demographic information including ethnicity, parental educational level, total monthly household income and medical history of their child prior to the study. Ethnicity was self-reported (Malay, Chinese, Indian, Others). 

Anthropometric measurements on the adolescents were performed by trained enumerators following a standard protocol. Body weight was measured in light clothing and with shoes removed, to the nearest 0.01 kg using a digital calibrated floor scale (SECA 813, Hamburg, Germany). Height was measured without shoes to the nearest 0.1 cm with a portable stadiometer (SECA 813, Hamburg, Germany). BMI was calculated in kg/m^2^ and was converted to standardised z-scores, adjusted for gender and age based on the International Obesity Task Force (IOTF) cut-off points [34]. Waist circumference was measured to the nearest 0.1 cm at the umbilicus, between the tenth rib and the iliac crest. Hip circumference was measured to the nearest 0.1 cm at the point of maximum extension of the buttocks using a flexible tape measure (SECA 203, Hamburg, Germany). The 90^th^ percentile was used as the cut-off point to identify abdominal obesity among Malaysian adolescents [35]. This translated to waist circumferences above 83.8 cm for boys and above 78.8 cm for girls, aged 13 years old.

### Statistical analyses

In Phase 1, EFA was conducted using the Principal Axis Factoring (PAF) extraction method with Promax (oblique) rotation to analyse the underlying structure of the Malay self-reported CEBQ and to determine if the structure was similar to the original CEBQ. Several criteria were used to determine the number of factors to retain including the eigenvalues > 1.0 rule and interpretation of the scree plot. More importantly, the factors retained had to be guided theoretically [36]. Barlett’s test of sphericity was used to examine if the variables were uncorrelated. Kaiser-Meyer-Olkin (KMO), a measure of sampling adequacy with values between 0 and 1 was used. Values between 0.50 and 0.70 were considered mediocre while values above 0.70 were considered good [37]. Only items with factor loadings of ≥ 0.32 were retained unless theoretically justified [32].

Reliability was assessed with internal consistency using Cronbach’s alpha coefficient, test-retest reliability (intraclass correlation coefficient (ICC)) and the split-half method. Correlations between subscales were determined using Pearson’s correlation coefficients. 

CFA was performed with AMOS version 20 to test the fit of the data using samples from Phase 2. A combination of several fit indices and their generally accepted values were used to assess good model fit. The indices included Chi-square/df ratio (less than 3.0), comparative fit index (CFI), Tucker-Lewis index (TLI), goodness-of-fit index (GFI), adjusted goodness-of-fit index (AGFI) values greater than 0.90 and the root-mean-square error of approximation (RMSEA) equals to or lesser than 0.70 [38]. While statistics guidelines must be considered, any modifications made to the model including decisions to retain or drop the item must be justified theoretically [39]. 

Weights, a multiplicative inverse of probability of selection, were applied to samples in Phase 2 to correct for unequal selection probabilities and non-response to produce unbiased estimates since multi-stage sampling was used. Gender and ethnic differences in eating behaviour were investigated with complex samples univariate analyses. Those of ‘Others’ ethnic (n = 21) were excluded from analyses on ethnicity due to small numbers. Complex samples GLM analyses examined associations between eating behaviour and BMI z-scores, adjusted for gender, ethnicity and maternal education level. Data were entered and analysed using SPSS version 21.0 (SPSS Inc., Chicago, IL, USA). All statistical tests were two-sided and significance level was set at p<0.05. 95% confidence intervals were reported.

## Results

### Phase 1

A total of 362 adolescents completed the Malay self-reported CEBQ in the validation study (Phase 1). Participants comprised of more males (59.7%) and were predominantly Malays (67.4%). 

### Factor structure

PAF with Promax rotation produced a nine-factor solution with eigenvalues above 1.0 which accounted for 54.15% of the total variance (Table 1). The Kaiser-Meyer-Olkin (KMO) value of 0.81 indicated sample adequacy. Bartlett’s test of sphericity indicated that the correlation between items were sufficiently large for this analysis (p<0.001). Most of the items loaded on the expected scales and were comparable with previous studies [13-16,20]. 

**Table 1:** Factor loadings on Promax Rotated Solution of Principal Axis Factoring of self- reported CEBQ items (n = 362).

**Scales**	**Loadings**	**Scales**		**Loadings**
**Food responsiveness (FR)**			**Emotional Overeating (EOE)**	
**(16.6% variance)**			**(4.5% variance)**	
I always want something to eat	0.42		I eat more when I am worried	0.62
I tend to eat more than I should	0.52		I eat more when I am annoyed	0.56
Given the choice, I would eat most of the time	0.60		I eat more when I am anxious	0.53
Even if I am full, I find room to eat my favourite food	0.54		I eat more when I have nothing else to do^(a)^	0.60
Given the chance, I would always have something to	0.31		**Enjoyment of Food (EF)**	
munch on			**(3.4% variance**)	
**Desire to Drink (DD)**			I love food	0.72
**(8.6% variance)**			I am interested in food^(a)^	0.48
I always want soft drinks	0.61		I look forward to mealtimes^(a)^	0.62
Given the chance, I would have soft drinks continuously	0.75		I enjoy eating	0.60
throughout the day			**Satiety Responsiveness (SR)**	
Given the chance, I would always have a soft drink	0.83		**(3.1% variance)**	
**Slowness in Eating (SE)**			I have a big appetite^(a)#^	0.53
**(5.7% variance)**			I get full easily	0.20
I finish my meals more quickly than others^#^	0.39		I cannot eat a meal if I have had a snack just before	0.32
I eat slowly	0.83		**Food Fussiness 1 (FF1)**	
I take a long time to finish a meal	0.65		**(3.3% variance)**	
I eat more and more slowly during the course of a meal	0.55		I am difficult to please with meals	0.41
**Emotional Undereating (EUE)**			I decide that I don’t like a food even without tasting it	0.42
**(5.3% variance)**			I leave food on my plate at the end of a meal^(b)^	0.61
I eat less when I am angry	0.79		I get full before I finish my meal^(b)^	0.27
I eat less when I am tired	0.38		**Food Fussiness 2 (FF2)**	
I eat more when I am happy^(a)^	0.27		**(3.7% variance)**	
I eat less when I am upset	0.50		I refuse to try new foods at first	-0.29
			I enjoy tasting new foods^#^	-0.60
			I enjoy a wide variety of foods^(a)#^	-0.53
			I am interested in tasting food I haven’t tasted before^#^	-0.60

^(a)^ These items loaded onto the FR scale but were retained on their original scales on theoretical grounds.

^(b)^ These items from the SR scale loaded on the FF1 scale.

^#^ reverse-scored items

An item on the FR scale, ‘I tend to eat more than I should’ split into a new factor but was retained on its original scale based on theoretical grounds. Four items, ‘I eat more when I am happy’ (EUE), ‘I eat more when I have nothing else to do’ (EOE), ‘I have a big appetite’ (SR) and ‘I enjoy a wide variety of foods’ (FF2) loaded on the FR scale, however, they have been provisionally retained on their original, theoretically determined scales. The EF scale also split into two factors, separating these pair of items, ‘I am interested in food’ and ‘I look forward to mealtimes’ from ‘I love food’ and ‘I enjoy eating’. The first pair of items loaded onto the FR scale but the EF scale was retained as a single factor for subsequent analysis. 

In the original CEBQ, the SR scale consisted of 5 items while the FF scale consisted of 6 items. The factor structure of the Malay self-reported CEBQ showed that the FF scale differentiated into two factors; FF1- dislike towards food (‘I am difficult to please with meals’ and ‘I decide that I don’t like a food even without tasting it’) and FF2- trying new foods (‘I refuse to try new foods at first’, ‘I enjoy tasting new foods’, ‘I enjoy a wide variety of foods’ and ‘I am interested in tasting food I haven’t tasted before’). Two items from the SR scale (‘I leave food on my plate at the end of a meal’ and ‘I get full before I finish my meal’) loaded on the same factor as FF1 and thus, were combined with that scale. The remaining three items on the SR scale were omitted from the study instrument as two of the items (‘I get full easily’ and ‘I cannot eat a meal if I have had a snack just before’) had low loadings. Moreover, a scale with less than three items is considered weak and unstable [40]. Hence, the remaining 8-factors (32-items) which differed from the original CEBQ were retained for further analyses. 

Samples from Phase 2 (n=646) were used in CFA to test the fit of the 8-factor model. The model was reduced by removing two items with low factor loadings (< 0.32), one at a time. These items included ‘I finish my meals more quickly than others’ (SE) and ‘I refuse to try new foods at first’ (FF1). The fit indices demonstrated that this model (8-factor, 30-items) had an improved albeit moderate fit (Chi-square/df = 3.686, CFI = 0.850, TLI = 0.815, GFI = 0.736, AGFI = 0.773, RMSEA = 0.065). The average variance extracted (AVE) values were greater than the R-squared values between the constructs, indicating sufficient discriminant validity. The composite reliability (CR) statistic for most scales approached 0.70, suggesting that most indicators were consistent in representing their respective latent constructs (results not reported here). Subsequent analyses were performed on the 30-items.

### Reliability

Cronbach’s alpha for each subscale of the self-reported CEBQ ranged from 0.48 to 0.76. (Table 2). Mean scores at Test 1 and Test 2 were calculated for each subscale (Table 2). Mean differences between both occasions were marginal. The intra-class correlation coefficient (ICC) showed good agreement on all scales, ranging from 0.72 to 0.90 (Table 2). The split-half method showed that the participants’ scores from the two halves of the questionnaire were moderately correlated (r = 0.546).

**Table 2:** Internal consistency (n = 362) and test-retest reliability (n = 133) of the self-reported CEBQ.

		**Average**				
		**corrected**			**Intraclass**	
		**item-total**			**correlation**	
	**Cronbach’s**	**correlation**	**Test 1**	**Test 2**	**coefficient**	
**Subscales**	**alpha**	**(range)**	**Mean (SD)**	**Mean (SD)**	**(ICC)**	**95%CI**
Food responsiveness (FR)	0.62	0.38 (0.28-0.43)	2.76 (0.85)	2.65 (0.82)	0.88	(0.83,0.92)
Enjoyment of food (EF)	0.70	0.49 (0.38-0.56)	3.26 (0.81)	3.20 (0.86)	0.90	(0.87,0.93)
Emotional overeating (EOE)	0.64	0.42 (0.33-0.47)	2.06 (0.79)	2.06 (0.82)	0.90	(0.85,0.93)
Desire to drink (DD)	0.75	0.59 (0.50-0.64)	2.56 (1.03)	2.49 (1.03)	0.87	(0.82,0.91)
Slowness in eating (SE)	0.71	0.53 (0.45-0.60)	2.40 (0.89)	2.36 (0.87)	0.84	(0.78,0.89)
Emotional undereating (EUE)	0.59	0.37 (0.27-0.46)	2.56 (0.87)	2.44 (0.83)	0.74	(0.63,0.81)
Food fussiness 1 (FF1)	0.48	0.28 (0.22-0.36)	2.44 (0.67)	2.41 (0.62)	0.73	(0.62,0.81)
Food fussiness 2 (FF2)	0.58	0.39 (0.30-0.44)	2.75 (0.93)	2.75 (0.93)	0.84	(0.78,0.89)

### Correlation between subscales

Correlations between the subscales presented in Table 3 indicated that the food-approach (FR, EF, EOE, DD) subscales and food-avoidant (SE, EUE, FF1, FF2) subscales were positively inter-correlated within their respective scales. Moderate correlations were found for FR-EF and FR-EOE compared to lower correlations reported for EF-EOE and EOE-EUE.

**Table 3:** Correlations between the self-reported CEBQ subscales (n = 362).

**Subscales**	**FR**	**EF**	**EOE**	**DD**	**SE**	**EUE**	**FF1**	**FF2**
Food responsiveness (FR)^δ^	**-**							
Enjoyment of food (EF)^δ^	0.54^**^	**-**						
Emotional overeating (EOE)^δ^	0.50^**^	0.37^**^	**-**					
Desire to drink (DD)^δ^	0.24^**^	0.16^**^	0.19^**^	**-**				
Slowness in eating (SE)^ε^	0.09	0.12^*^	-0.06	0.16^**^	**-**			
Emotional undereating (EUE)^ε^	0.26^**^	0.16^**^	0.35^**^	0.18^**^	0.29^**^	**-**		
Food fussiness 1 (FF1)^ε^	0.25^**^	0.16^**^	0.22	0.14^**^	0.26^**^	0.33^**^	**-**	
Food fussiness 2 (FF2)^ε^	-0.41^**^	-0.42^**^	-0.26^**^	-0.14^*^	-0.02	-0.19^**^	-0.11^*^	**-**

^δ^ food-approach subscales, ^ε^ food-avoidant subscales, ** p < 0.01 , * p < 0.05 (two-sided).

### Phase 2

A total of 646 adolescents completed the questionnaire. Six cases were excluded due to missing data and outliers. Approximately three-quarters of the sample were Malays. The average waist circumference was in the normal range. About 27% of the adolescents were identified as overweight or obese. Close to half of the parents completed secondary school education while about 20% possessed tertiary education (Table 4).

**Table 4:** Descriptive characteristics of Phase 2 sample (n = 646).

	**N**	**Weighted %**
Gender		
Male	182	26.8
Female	464	73.2
Ethnicity		
Malay	493	71.8
Chinese	78	15.9
Indian	54	9.6
Others	21	2.7
Weight (kg)^#^	47.49 (14.17)	
Height (cm)^#^	151.08 (7.70)	
Waist circumference (cm)^#^	68.64 (12.45)	
Body mass index (BMI)^#^	20.58 (5.06)	
BMI z-score^#^	0.21 (0.93)	
BMI categories		
Underweight	52	8.0
Normal weight	422	65.3
Overweight	104	16.1
Obese	68	10.5
Monthly household income (RM)^#^	2696.19 (3404.56)	
Household income brackets (RM)		
499 and below	173	29.3
500 – 999	41	5.0
1000 – 1499	40	5.8
1500 – 1999	64	8.5
2000 – 2499	41	6.8
2500 – 2999	43	5.9
3000 – 3499	41	6.4
3500 – 3999	21	3.2
4000 – 4999	40	5.6
5000 and above	111	23.5
Father’s education level		
Primary	36	6.4
Secondary	266	41.2
Tertiary	115	19.5
Mother’s education level		
Primary	43	7.8
Secondary	294	44.9
Tertiary	102	17.6

^#^ mean (SD)

### Gender and ethnic differences

**Figure 2:**
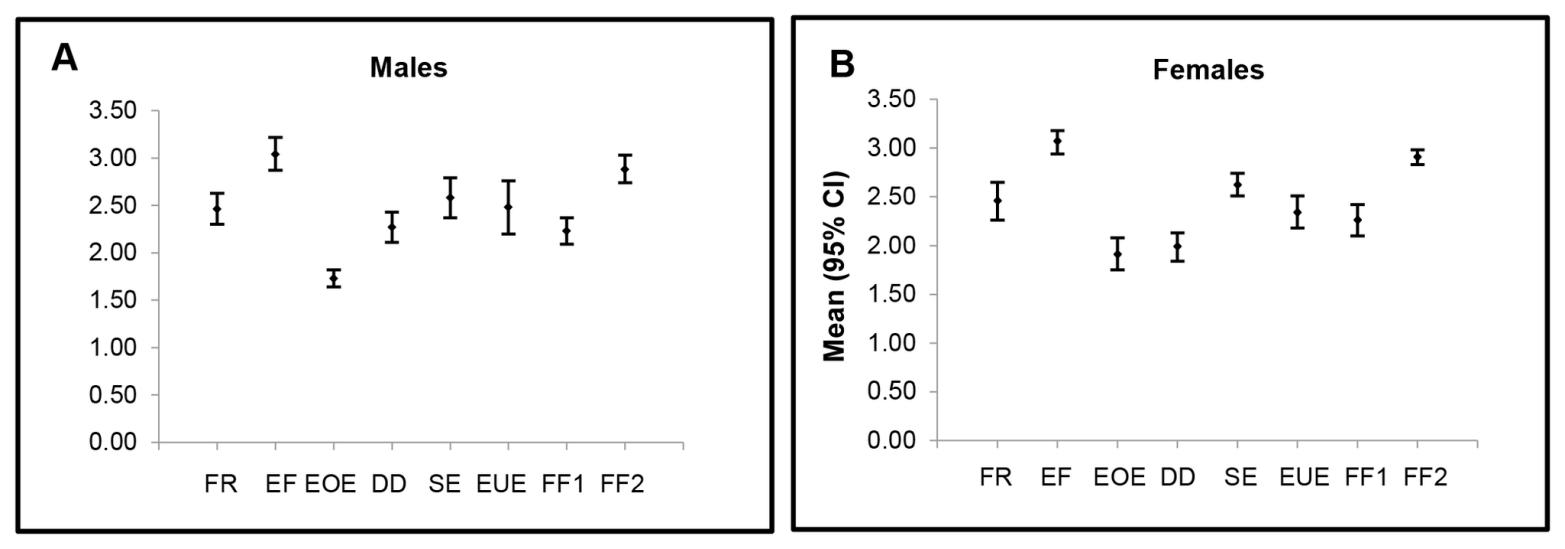
Eating behaviour mean scores of 13 year old Malaysian adolescents by gender. Error bars indicate 95% CI. Mean scores were obtained with the self-reported CEBQ. Statistical significance was not detected.

**Figure 3:**
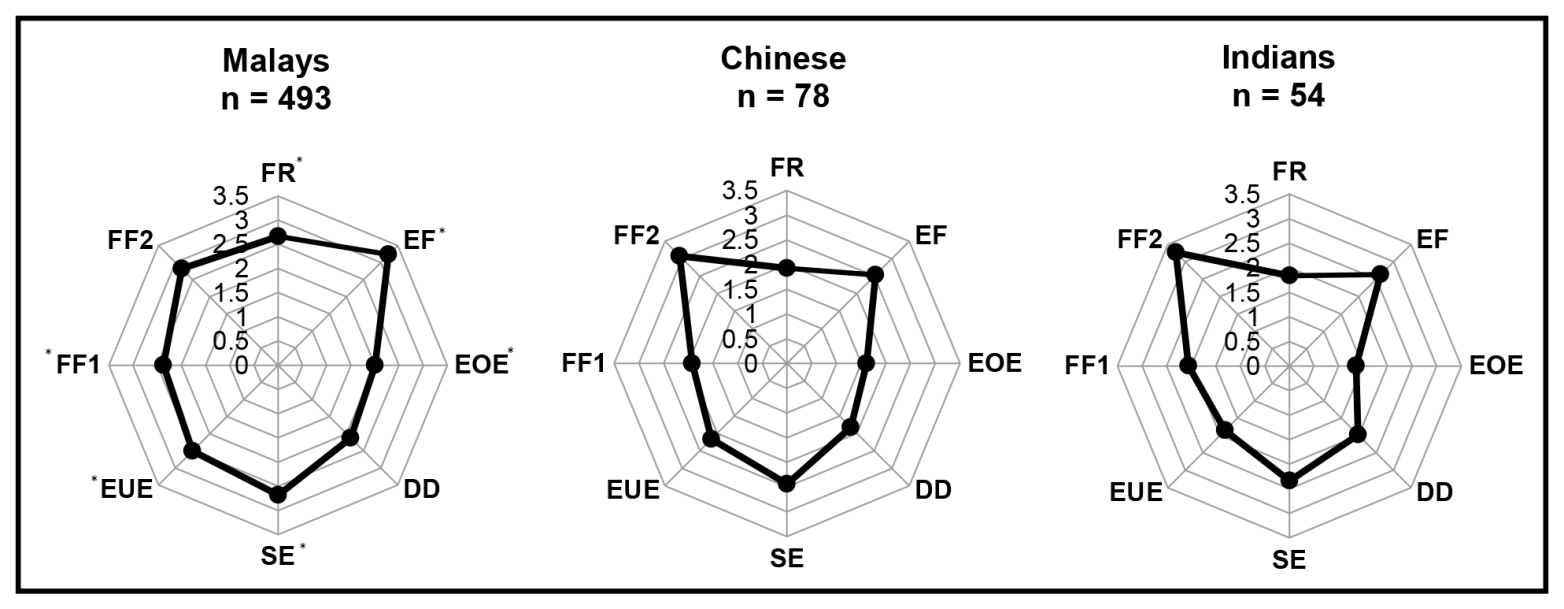
Eating behaviour mean scores among 13 year old Malaysian adolescents by ethnicity. (Clockwise) The four ‘food-approach’ subscales are indicated along the polygons followed by the four ‘food-avoidant’ subscales. Markers indicate point estimates of the eating behaviour scores on the self-reported CEBQ among the Malay, Chinese and Indian adolescents. ^*^ Statistical significance.

Statistical significance in eating behaviours was not detected between genders (Figure 2). Ethnic differences in eating behaviours among the three major ethnic groups are graphically represented in individual radar charts (Figure 3). The four food-approach subscales were indicated along the points of the polygon in clockwise direction followed by the four food-avoidant subscales. Malay adolescents reported significantly higher FR, EF, EOE, SE, EUE and FF1 mean scores compared to their Chinese and Indian peers.

### Associations between eating behaviour and BMI z-scores

The FF1 scale (‘dislike towards food’) was found to be negatively significant with BMI z-scores when adjusted for gender, ethnicity and maternal educational level (Table 5).

**Table 5:** General linear model analyses of the self-reported CEBQ subscale scores and BMI z-scores (n = 646).

	**Adj. mean**			
**Subscales**	**(95% CI)**	**β (95% CI)**	**R^2^**	**F (df1, df2), p**
Food responsiveness (FR)	2.46 (2.29, 2.63)	-0.03 (-0.10, 0.05)	0.07	F (1,6) = 0.64, p = 0.46
Enjoyment of food (EF)	3.06 (2.94,3.17)	-0.02 (-0.09, 0.05)	0.07	F (1,6) = 0.62, p = 0.46
Emotional overeating (EOE)	1.87 (1.73, 2.01)	0.03 (-0.03, 0.09)	0.07	F (1,6) = 1.36, p = 0.29
Desire to drink (DD)	2.06 (1.93,2.18)	-0.11 (-0.22, 0.00)	0.08	F (1,6) = 0.60, p = 0.05
Slowness in eating (SE)	2.61 (2.51, 2.71)	-0.06 (-0.20, 0.09)	0.07	F (1,6) = 0.95, p = 0.37
Emotional undereating (EUE)	2.38 (2.27, 2.48)	0.04 (-0.04, 0.11)	0.07	F (1,6) = 1.47, p = 0.27
Food fussiness 1 (FF1)	2.25 (2.12, 2.39)	-0.12 (-0.23, -0.02)	0.08	F (1,6) = 7.93, p = 0.03^*^
Food fussiness 2 (FF2)	3.02 (2.83, 2.97)	-0.05 (-0.12, 0.02)	0.07	F (1,6) = 3.04, p = 0.13

^*^ p < 0.05; GLM complex samples adjusted for gender, ethnicity and maternal education level.

## Discussion

This present study pioneers the adaptation of the CEBQ as a self-report among adolescents compared to the original parent report for toddlers and younger children used in previous studies. To the best of our knowledge, our study is the first to validate an instrument that investigates eating behaviours associated with weight gain among multi-ethnic urban Malaysian adolescents.

In our sample, a nine-factor structure solution explained 54% of the variance. The SR scale was dropped due to low-loading items. CFA supported the 8-factor model which contained variations from the original CEBQ. The four items from different subscales that loaded on the FR scale were retained on their original, theoretically determined scales. The EF scale split into two factors, separating these pair of items, ‘I am interested in food’ and ‘I look forward to mealtimes’ from ‘I love food’ and ‘I enjoy eating’ but was retained as a single factor for subsequent analysis. Enjoyment of food can be attributed to the allure of taste, aroma, appearance and variety of food available. These make eating a pleasurable experience such as sharing conversations over a gastronomical delight. That said, individuals have an innate preference for energy-dense foods that are more satiating which thereby, increases overall energy intake [41]. 

An interesting finding was that the adolescents perceived food fussiness as two different concepts; dislike towards food (FF1) and trying new food (FF2). A picky or fussy eater is often someone who is selective of foods, consumes an inadequate variety of food [42] and therefore, is at risk of nutritional deficiency. Food preferences commonly involve sensory properties including taste, texture, colour, appearance and aroma [43]. Rozin and Fallon (1987) postulated three motivational dimensions for acceptance or rejection of foods – anticipated consequences (danger), sensory perception of food (distaste) and ideational reaction towards food (disgust) [44]. Thus, an individual resolves a dislike towards a certain food not when the food is tasted rather when the food is within sight. The FF2 scale comprised of items that revolve around trying new foods. Food neophobia refers to the reluctance to eat or the avoidance of new foods [19]. Similar to food rejection, willingness to try novel foods can be decided solely on visual cues. Previous negative experiences and unfamiliarity can likewise invoke a cautionary response. 

Two items from the SR scales clustered with the FF1 items and therefore, were combined into the FF1 scale. Reaching or displaying early satiety and not having a clean plate may possibly be related to being a picky eater, showing distaste or disgust or perceived harm. Leaving food on the plate at the end of a meal may be regarded as being wasteful and is culturally frowned upon due to superstitious beliefs and seen as disrespect or lack of gratitude in the local culture [45]. The remaining items on the SR scale were omitted due to low factor loadings. It is plausible that the adolescents could not identify with these items.

The psychometric properties of the Malay self-reported CEBQ (30-items, 8-factor) were generally acceptable. Most scales showed strong internal consistency similar to previous works [13,14,16,20,22] except for EUE, FF1 and FF2 scales. The extent to which an individual experiences hyperphagia (overeating) or hypophagia (undereating) during emotional distress may vary and evidence is still inconclusive [11]. Cultural beliefs and norms towards being fussy about food should be given due consideration. Exhibiting satiety before finishing a meal and food fussiness is often regarded as discontentment in our local culture. 

The self-reported CEBQ demonstrated good reliability between the two administrations. Prior to this, a test-retest was performed only with the original CEBQ and was analysed using Pearson’s correlation coefficient [13]. The test on the second occasion was intentionally administered under conditions as similar as possible to the first test. Several factors such as the interval between Test 1 and Test 2, recall bias, true changes and intrinsic feelings must be acknowledged as these may have potentially affected test-retest reliability. In addition, a moderate correlation resulted from the split-half method indicated an acceptable level of reliability. 

The four food-approach scales were positively correlated as each scale independently and collectively measured increased appetitive traits. Unlike prior findings whereby EF, EOE, FR, SR, SE and FF scales differed significantly between genders [15,16,21], no statistically significant differences in eating behaviour were detected between the male and female adolescents in our study. This is interesting and warrants further investigation since female adolescents; in particular, tend to exhibit weight concerns which often results in dietary restraint or restrictive eating. Adolescents are also vulnerable to eating disorders often fuelled by negative body image perceptions or low self-esteem [19]. It is worth mentioning that a trend of EF, EOE and SE was observed among female adolescents, similar to Santos and colleagues [15]. Food-approach behaviours may predispose them to a higher risk of developing obesity. This interest in eating should be nurtured towards self-efficacy in the preparation, selection and consumption of healthy eating. The food-avoidant behaviours identified including sensitivity to satiety signals and a healthy eating pace should be encouraged to prevent unnecessary weight gain among these adolescents. 

Comparison of eating behaviours across ethnicities among Malaysian adolescents using the CEBQ is previously unexplored. Food responsiveness, enjoyment of food, emotional overeating, slowness in eating, emotional undereating and food fussiness 1 (‘dislike towards food’) were most significantly reported among the Malay adolescents. Malays originated from across the South East Asian archipelago which infuses diverse and rich flavours into their cooking. Malay cuisine, in particular, is very aromatic, flavourful and comprises of a delectable variety. Within Peninsula Malaysia, traditional Malay food is recognised by their states, each with their unique taste and cooking styles [46,47]. It is possible that growing up eating flavourful foods may have developed strong taste preferences. Moreover, Malays, in general, are very communal, collectivist and hospitable in nature. With the mushrooming of eateries and 24-hour ‘mamak’ (Indian Muslim) restaurants, the accessibility and availability to an abundance of food is unrivalled. In that vein, eating in a hurried pace is discouraged as it equates to gluttony. This may elucidate the relaxed pace of eating observed among these adolescents. This is speculative but worthy of future research to investigate the appetitive traits observed among this ethnic group. 

Findings in this study surfaced the influence of emotions on eating behaviours significantly observed among Malay adolescents. Emotional stress has found to increase or decrease eating responses. Emotional eaters or hyperphagics regulate or escape negative emotions by overeating, often sweet, high-fat and energy-dense foods which increases the risk of adiposity [48]. Conversely, intense emotional arousal can reduce gut activity, as observed in emotional undereating or hypophagia [48]. Stemming from a high-context culture that involves emotions and values relationships, the Malays emphasise manners or ‘adab’, propriety and prefer indirect, non-verbal communication. Anthropological studies concur that they are generally polite, obliging, considerate towards the feelings of others and strive to preserve harmony [49,50]. Overeating when experiencing negative emotions may be regarded as a non-disruptive coping mechanism. However, triangulation of data is needed and more work is advisable to elucidate and confirm this finding. Guiding these adolescents on how to channel their emotions into constructive activities is strongly recommended. Empowering these adolescents with strategies to manage ‘hedonic hunger’, particularly in an ‘obesogenic’ environment is likewise necessary to avoid overindulgence in food intake. 

Several authors have found that BMI z-scores were positively associated with FR, EF and EOE and were inversely associated with SR and SE scales [14-16] while others have reported no associations [20,21]. We found BMI z-scores were inversely associated with FF1 (‘dislike towards food’). Obese individuals tend to be less fussy eaters compared to their leaner counterparts, however, previous studies have yielded mixed results [14,15]. While food fussiness may appear to be protective against excessive calorie intake and subsequent weight gain, a well-balanced and nutritionally-adequate diet should not be compromised. 

Possible reasons that our findings differ from the original CEBQ are three-fold. Firstly, the parent report may reflect the parent’s perception of their child’s eating behaviour and this definitely varies from an adolescent’s self-evaluation of their own eating styles. Secondly, while age difference was not the focus of this study, it should be highlighted that the parent report focused on younger children whereas studies among adolescents were unexplored prior to this. Thirdly, cultural differences in a multi-ethnic Asian setting should be considered since the influence of culture is complex and is mediated by biological, demographic, psychosocial and environmental factors [51]. 

The sample in Phase 2 (association study) may have under-represented the Chinese ethnic group in the country possibly due to volunteer bias. Causality cannot be determined due to the cross-sectional design. It is recommended to replicate this study in a more heterogeneous sample with a wider age range.

Despite these limitations, this study spearheads the validation of the CEBQ as a self-report measure to investigate eating styles related to obesity among adolescents of various ethnicities in Malaysia. Understanding behavioural traits allows for planning and implementation of obesity prevention and intervention strategies in the local context. This self-report questionnaire can then be used further as an assessment tool to evaluate the effectiveness of those measures. 

## Conclusions

Although CEBQ is a valuable psychometric instrument, adjustments were required due to age and cultural differences in our sample. While previously used as a parent-report among younger children, the self-reported CEBQ now empowers adolescents to evaluate their own eating behaviours from a range of dimensions. Our findings reveal the influence of gender, ethnicity and weight status on eating behaviour amongst multi-ethnic Malaysian adolescents using the Malay self-reported CEBQ. In addition, a lack of dislike towards food was observed among obese adolescents. Future longitudinal and qualitative studies are warranted to understand behavioural phenotypes of obesity and to guide prevention and intervention strategies. 
